# Error management climate, psychological security, and employee bootleg innovation behavior: the moderating role of risk-taking traits

**DOI:** 10.3389/fpsyg.2025.1538584

**Published:** 2025-03-17

**Authors:** Qing Wang, Xiaoli Zhang, Na Zhang, Jiafu Su

**Affiliations:** ^1^Anhui Technical College of Mechanical and Electrical Engineering, Wuhu, China; ^2^College of Mechanical and Vehicle Engineering, Chongqing University, Chongqing, China; ^3^International College, Krirk University, Bangkok, Thailand

**Keywords:** bootleg innovation behavior, error management climate, psychological security, risk-taking traits, creativity

## Abstract

Employee bootleg innovation behavior is the key to helping enterprises get rid of the “innovator’s dilemma” and achieve innovative development. This article constructed a model of the relationship between error management climate, psychological security, risk-taking traits, and employees’ bootleg innovation behaviors based on social cognitive theory and tested the model empirically. The results show that error management climate has a significant positive influence on employees’ bootleg innovation behavior; psychological security plays a mediating role between error management climate and bootleg innovation behavior; and risk-taking traits play a moderating role in the relationship between psychological security and employees’ bootleg innovation behavior. The results of the study provide valuable insights for guiding employees’ bootleg innovation behaviors and help organizations in effectively managing these behaviors, thus enhancing organizational innovation performance.

## 1 Introduction

With the rapid development of the global economy and the unpredictable social market environment, innovation has increasingly become the core competitiveness of countries and enterprises. Whether it is a country or an enterprise, only through continuous innovation and change can it continuously increase its value and gain a stronger competitive advantage (Farida and Setiawan, 2022). National innovation relies on firm innovation, which is driven by the innovative ideas of individuals and ultimately realized through their innovative behavior (Wang and Bai, 2020). Therefore, for countries and enterprises to achieve innovation goals, it is crucial to effectively motivate and guide the innovative behaviors of employees involved in management, research and development, production, sales, and other areas within the enterprise. However, there is a common paradox in the current practice of organizational innovation: organizations will give employees a high degree of work autonomy to stimulate their willingness to innovate, but strict rules and regulations and management processes within the organization will constrain their innovative behaviors. Gilson (2024) states that when employees are expected to be creative but are not in a position to achieve innovative behavior, the only way to creativity is to break the established rules, i.e., to innovate informally, in a bootleg manner. Bootleg innovation is an innovative activity initiated by employees autonomously from the bottom up, without formal support from the organization, unknown to top management, and expected to contribute to enhancing the interests of the organization, although contrary to organizational norms or regulations (Criscuolo et al., 2014). Bootleg innovation behavior is closely related to innovation performance. Employees with bootleg innovation behavior are able to actively search for the required resources, extend their working hours, show more creativity and initiative, and thus have a positive impact on innovation performance (Wang and Wan, 2020). It was found that 63.6% of employee bootleg innovation behavior leads to product innovation and 10.9% of bootleg innovation behavior promotes corporate knowledge creation and learning (Masoudnia and Szwejczewski, 2012). Therefore, employees, as an important force for change in corporate development, are highly likely to help companies escape from the “innovator’s dilemma” and achieve better development through bootleg innovation for the purpose of promoting organizational development.

As employee bootleg innovation behavior plays an important role in improving organizational innovation performance, scholars have conducted a large number of studies around employee bootleg innovation behavior, among which the exploration of its formation mechanism is the most abundant. There is an interaction between environment, individual, and behavior, in which the environment plays a guiding role in behavior (Bandura and Walters, 1977). Error management climate as one of the organizational cultures can have a profound effect on the bootleg innovation behavior of employees. Error management climate refers to the perception of errors held by the organization as well as the processes and behavior associated with the organization’s handling of errors as perceived by employees (Yang et al., 2024). In essence, bootleg innovation behavior is a kind of trial-and-error learning behavior. Due to limited information, knowledge, and ability, coupled with a lack of organizational support, employees are likely to make mistakes while implementing bootleg innovation behaviors. These behaviors are largely affected by how the organization handles these mistakes. If organizations and managers focus on creating an error management climate in their daily management, fostering a culture that tolerates mistakes, encourages trial and error, and supports innovation, it can help alleviate employees’ fear of making errors and encourage them to step out of their comfort zones in the pursuit of innovation. This environment will make employees take risks, innovate, and engage in more innovative behaviors. Therefore, in order to more effectively manage the staff’s innovative behavior and promote their contribution to organizational innovation and change, it is necessary to enhance the understanding of the deep-rooted mechanisms and effects of the staff’s innovative behavior under the error management climate.

When employees consider implementing bootleg innovation behavior, given the high risk of the behavior, they go through a crucial stage of psychological weighing, carefully evaluating the potential benefits and potential losses that the behavior may bring (Huang et al., 2022). An error management climate creates a work environment where errors are treated positively and can lead to a high level of psychological security for the individual (Maqsoom et al., 2023). Psychological security, as a positive psychological perception, can effectively reduce the depletion of psychological resources for employees to make bootleg innovation behavior. Therefore, psychological security may play a mediating role between error management climate and employees’ bootleg innovation behavior. In addition, as bootleg innovation behavior require employees to hide their behavior from the organization and superiors, or to carry out activities against the orders of the organization and superiors (Criscuolo et al., 2014). Therefore, bootleg innovation behavior is more likely to require the addition of employee risk-taking traits than general innovation behavior. Employees with higher risk-taking traits have higher motivation for bootleg innovation drive violation and thus are more likely to engage in bootleg innovation behavior. In view of this, this paper intends to introduce risk-taking traits as a moderating variable to unravel the influence mechanism between psychological security and employee bootleg innovation.

To sum up, based on the theoretical framework of social cognition, this paper starts from two dimensions of error management climate and psychological security, integrates the consideration of employees’ risk-taking traits, and follows the logical path of “scene-cognition-behavior” to deeply explore the specific impact of error management climate on employees’ bootleg innovation behavior. This study not only helps deepen the understanding and discussion of the theory of bootleg innovation behavior but also has important practical significance for reasonably stimulating employees’ innovation potential and effectively guiding enterprises to build scientific innovation management systems.

## 2 Literature review and hypotheses development

### 2.1 Error management climate and bootleg innovation behavior

An error management climate is the creation of a cultural environment in an organization or team that encourages employees to report errors, share lessons learned, and improve work processes (Jia et al., 2023). It emphasizes open acceptance of mistakes and positive responses to facilitate learning and continuous improvement. In this cultural environment, employees are more likely to engage in bootleg innovation behavior (Liang et al., 2023). On the one hand, this is because organizations with a good error management climate actively confront errors, rather than blindly rejecting them, which reduces employees’ fear of taking risks and trying new approaches, and encourages them to be more willing to try out new ideas and approaches, which, in turn, stimulates employees’ potential for innovation and makes them willing to engage in challenging work (Geng et al., 2022b). On the other hand, organizations with a fault-tolerant atmosphere regard errors as learning opportunities, encourage employees to report errors, share lessons learned, and discuss improvement measures together. This culture encourages employees to communicate more willingly when facing errors, to pass on information about errors, and to discuss solutions together, so that they can share and learn from error knowledge, accumulate error experience, and improve their ability to detect and correct errors (Geng et al., 2022a). In summary, the organization’s encouragement, tolerance, and support for employees in an error management climate not only alleviates employees’ concerns about failure and risk but also makes them more willing to share mistakes and lessons learned, and inspires them to be bolder in exploring new territories and engaging in bootleg and innovative behaviors. In view of the above analyses, this study proposes the following hypotheses:

*H1: Error management climate positively influences employee bootleg innovation behavior.*

### 2.2 Mediating role of psychological security

Organizations with a good error management climate have a high degree of tolerance for errors, are tolerant of those who make mistakes, motivate them to make progress, and advocate employees to communicate with each other, learn from each other, and continuously improve and perfect their own abilities. Psychological security refers to the staff’s perception of the degree of safety in their surrounding environment. A positive error management climate can effectively enhance the level of psychological security among staff. The higher the employee’s sense of psychological security, the more they are not afraid of difficulties, the more courageous they are to challenge, and the more out-of-role behaviors they show (Zheng et al., 2024). The error management climate focuses on opportunities to improve employee workflow rather than the negative consequences of work errors. It encourages employees to share rather than cover up information about mistakes, which gives employees confidence that they will not be mocked and punished for making mistakes, reducing the risk they may take for making mistakes at work, resulting in a higher level of psychological security (Zhao et al., 2023). In addition, the error management climate encourages employees to communicate openly with other members of the organization when errors occur, and this open communication of errors allows employees to learn not only from their own errors but also from the sharing of errors with others, which enhances the understanding and connection between employees, forming a trusting and mutually supportive interpersonal relationship (Zhang et al., 2024). This supportive work environment not only meets the instrumental resource needs of employees but also meets the socio-emotional resource needs of employees, so that employees can psychologically experience a sense of security in working in the organization (Agarwal and Farndale, 2017). In view of the above analysis, the following hypotheses were formulated for this study:

*H2: Error management climate positively affects employees’ psychological security.*

Psychological security can enhance employees’ perceptions of organizational inclusiveness on the one hand, and on the other hand, it can circumvent the emergence of employees’ worrying emotions, thus daring them to pursue their personal values and goals. Bootleg innovation as a challenging extra-role behavior may also be influenced by psychological security. Bootleg innovation as an extra-role behavior is usually carried out clandestinely behind the backs of the organization or superiors and carries a certain amount of risk (Liu et al., 2024). In general, employees who engage in exploratory risk-taking activities are usually affected by risk perception (Elsayed et al., 2023). However, a good sense of psychological security can lead to employees believing that it is safe to implement challenging behaviors in organizations and that they are willing to continue to learn, change, and innovate in complex and high-risk work environments (Edmondson et al., 2016). When employees have a high sense of psychological security, they can express their ideas and opinions freely without any constraints and without worrying about their status, position, etc. being affected (Kyambade et al., 2024). As a result, the perceived risk of bootleg innovation can be reduced, whereas employees with a low level of psychological security tend to avoid bootleg innovation because they are concerned that it is illegal and involves a high level of risk. In addition, when employees have a high level of psychological security, they will worry less about their own behavior and are willing to put forward more innovative ideas for the enterprise; on the contrary, when employees have a low level of psychological security, they will worry about whether their words and deeds are appropriate and safe, and will often be “tied up,” not daring to show themselves too much. They tend to be “tied up” and do not dare to express themselves too much. In conclusion, the following hypotheses are proposed in this study:

*H3: Psychological security positively influences employee bootleg innovation behavior.*

Eivazzadeh and Nadiri (2022) proposed that psychological security is a level of perception in which individuals are not worried about being affected by unfavorable factors in their surroundings and are able to express their thoughts truthfully and display different behaviors. It promotes employee transgression and innovation. Employees in an organizational environment with a good error management climate will perceive a higher level of psychological security, which will motivate them to initiate bootleg innovation as the perception of an organizational safety climate will enable them to overcome their fear of the unknown and to actively engage in self-accepted work behavior (Frazier et al., 2017). Furthermore, when the sense of psychological security is high, employees will believe that they will not be punished by the organization even if they make mistakes when implementing bootleg innovation behavior, and be sure that implementing challenging behavior is safe in the organization, and their focus will be on how to innovate, rather than fearing the risks, and then, they will be willing to continue to learn and innovate in their work; when the sense of psychological security is low, employees will be worried that the bootleg innovation behavior will not be able to achieve the expected results, and they will be repulsed by the consequences of the subsequent mistakes they will need to bear and the interpersonal risks, and they will refuse to break the rules in order to transgress the rules and innovate (Hood et al., 2016). Based on this, Hypothesis 4 is proposed in this study.

*H4: Psychological security mediates the relationship between error management climate and employee bootleg innovation behavior.*

### 2.3 The moderating role of risk-taking traits

Bootleg innovation behavior requires employees to resist social pressures and deviate from norms (Yang et al., 2024). Moreover, although bootleg innovation behavior aims to enhance the wellbeing of the organization and the employees’ intentions are good, its results are uncertain and may lower the image of the employees in the organization or it may invite resentment and rejection from others. Therefore, bootleg innovation behavior carries some risks. It is argued that risk-taking behavior is determined by the decision maker’s perceived risk and attitude toward the perceived risk (Jochemczyk et al., 2017). Risk-taking traits are an individual’s tendency to take or avoid risks and describe an employee’s attitude toward perceived risk. Risk-taking traits as an employee’s assessment of risk and reward can influence an employee’s expectations of outcomes (Rahim et al., 2024). It can be a good explanation of risky behavioral differences between individuals and can serve as a boundary condition for psychological security to effectively motivate employees to commit constructive bootleg behavior. When employees are low in risk-taking traits, risk-avoidant individuals may focus too much on negative consequences (Pearsall et al., 2023). For example, they may worry about the ridicule and isolation they may receive if they break the rules, they may worry that their behavior will be seen as a show, and they may worry that they will be seen as an outlier, so these individuals prefer to follow the rules. With low risk-taking traits, employees may choose not to act even if they possess a strong sense of psychological security, preferring not to excel and ensuring conformity. They may exaggerate the likelihood of loss and worry that a failed outcome will ruin everything, while ignoring potential gains. Out of concern for negative outcomes, they are thus reluctant to make bootleg innovations (Choma et al., 2014). On the contrary, when employees are high in risk-taking traits, individuals who tend to take risks are subjectively more concerned about the benefits of bootleg innovation behavior for the organization and other members, are willing to invest significant resources in developing opportunities or engaging in behavior with uncertain outcomes, and are willing to engage in the behavior even if the bootleg innovation poses risks and may be misinterpreted (Jia et al., 2021). Individuals high in risk-taking traits amplify factors that favor risky behavior and tend to arrive at a higher likelihood of success in the face of risky behavior than the norm. Individuals high in risk-taking traits have fewer concerns when making risky decisions and appear bolder. In short, risk-taking traits are individuals’ perceptions and perceptions of risk and opportunity. At the same time, employees’ concerns about risk lead to conservative behavior, while the emphasis on returns leads to the courage to break the rules (Luo et al., 2024). In view of the above analyses, this study proposes the following hypotheses:

*H5: Risk-taking traits positively moderate the relationship between psychological security and employees’ bootleg innovation behavior.*

**Figure 1:**
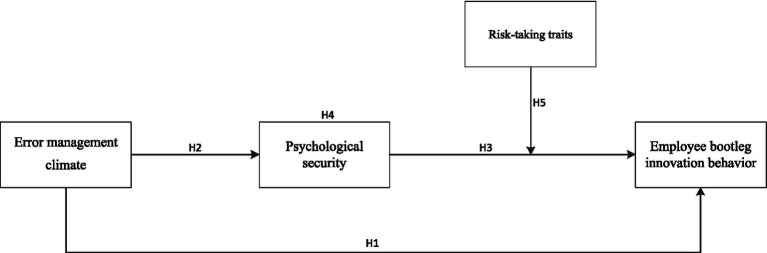
Conceptual model.

Through the variables designed in this paper and the summary of related literature, the interrelationships between error management climate, psychological security, risk-taking traits, and employees’ bootleg innovation behavior are analyzed based on social cognitive theory, and the following theoretical model is proposed in this paper (Figure 1).

## 3 Research design

### 3.1 Questionnaire design

In this paper, the research hypotheses are verified by means of a questionnaire survey. The target of the survey is the working staff of enterprise units in Beijing, Dalian, Yantai, and Taiyuan and involves different industries and positions such as finance, manufacturing, IT, real estate, and so on. As the capital, Beijing has a high degree of economic, cultural, and political influence. Dalian is an important seaport city located in northeast China. Yantai, located in the Shandong Peninsula, is an open coastal city. Taiyuan, the capital of Shanxi Province, has the characteristics of an inland city. These four cities differ in geographical location, economic development level, cultural background, and other factors, which helps enhance the regional representativeness of the study. At the same time, finance, manufacturing, IT, real estate, and other industries occupy an important position in the national economy, and each has a different operation model, working environment, and personnel quality. Choosing these industries as research objects can fully reflect the commonalities and differences of staff in different industries. In the process of questionnaire collection, we follow the principle of voluntariness and anonymity and do not affect the normal life of the respondents. At the same time, strict data protection measures are in place to ensure that participants’ answers are not disclosed to third parties during the collection, storage, and analysis process. The research was mainly carried out through the distribution of electronic questionnaires; first, 105 questionnaires were distributed for pre-survey, which were used for the initial questionnaire variables such as the reliability test, the results show that a coefficient is greater than 0.8, and the questionnaire reliability is good. After that, the formal research was carried out, in order to ensure the quality of the questionnaire, one-on-one explanation of the research problems and precautions will be given when the questionnaire is distributed online. A total of 282 electronic questionnaires were collected and screened. First, according to the response time of the questionnaires, by analyzing and discussing the response time of the sample as a whole, it was considered that the response time of a valid questionnaire should be more than or equal to 100 s, so questionnaires with a response time less than this criterion were treated as “did not fill out the questionnaire carefully.” Second, the questionnaires that all chose the same option or showed a clear pattern in their responses were deleted. Finally, 241 valid questionnaires were selected, with a validity rate of 85.46%.

The Statistical Package for the Social Sciences (SPSS) offers a wide range of data analysis functions, including descriptive statistics, inferential statistics, non-parametric testing, analysis of variance, regression analysis, correlation analysis, cluster analysis, factor analysis, and structural equation modeling. Therefore, this paper uses SPSS to process and analyze the data.

### 3.2 Demographic characteristics of participants

Gender differences may lead to differences in employees’ innovation motivation, risk appetite, and innovation methods. For example, some studies suggest that male employees may be more inclined to take risky and innovative behaviors, while female employees may be more detail-oriented and robust. These differences may affect whether employees choose deviant innovation behavior and the specific performance of their behavior. Age may affect an employee’s ability and willingness to innovate. Young employees usually have a stronger willingness to innovate and take risks and are more willing to try new ideas and methods; older workers, on the other hand, may be more focused on stability and experience accumulation and may be more cautious about bootleg innovation. Education level is usually closely related to employees’ knowledge reserve, learning ability, and innovation ability. Length of service may affect employee loyalty to the organization, familiarity with the job, and decision-making processes for innovative behavior. At the same time, the enterprise size may affect the innovation atmosphere, resource input, and management style of the organization. Large enterprises may have stronger innovation strength and better innovation systems, but they may also have problems such as slow decision-making and bureaucracy. Small businesses may be more flexible and innovative but have limited resources. These differences may influence employees’ bootleg innovation behavior. Therefore, we use the above five variables as demographic variables. The results of the analysis of the basic information of the sample show that 48.96% of all subjects were male and 51.05% were female; the proportion of those under 35 years of age amounted to 63.07%; the proportion of those with a bachelor’s degree or higher was 54.77%; the proportion of those who had worked for less than 3 years was 79.67%; and the proportion of those who came from enterprises of a size of less than 500 employees was 55.60%. Table 1 summarizes the demographic information of the sample of subjects in this study.

**Table 1:** Demographic characteristics of participants.

	Options	Frequency	Percentage	Cumulative percentage
Sex	Male	118	48.963	48.963
	Female	123	51.037	100
Age	18–25 years	75	31.12	31.12
	25–35 years	77	31.95	63.071
	35–45 years	63	26.141	89.212
	45–55 years	19	7.884	97.095
	55 years and over	7	2.905	100
Education	Undergraduate or below	109	45.228	45.228
	Undergraduate	81	33.61	78.838
	Bachelor’s degree	41	17.012	95.851
	Doctoral degree	10	4.149	100
Length of service	Less than 1 year	59	24.481	24.481
	1–3 years	133	55.187	79.668
	4–10 years	32	13.278	92.946
	11–25 years	12	4.979	97.925
	More than 25 years	5	2.075	100
Enterprise size	Less than 100	32	13.278	13.278
	100–500 persons	102	42.324	55.602
	500–1,000 persons	97	40.249	95.851
	More than 1,000 people	10	4.149	100
Total		241	100	100

### 3.3 Measurement of variables

The survey measured four variables: error management climate, psychological security, risk-taking traits, and bootleg innovation behavior, and the scales chosen were all mature scales. The scale was based on a 5-point Likert scale, with scores ranging from 1 to 5, indicating “not at all compliant” to “fully compliant.”

#### 3.3.1 Error management climate

Combined with the relevant theories of error management, and based on the research of Zhang (2021), this paper constructs an error management climate measurement scale, with a total of seven items, including “the enterprise has relevant error prevention measures,” “the enterprise has a sound error management system and regulations,” and so on. Cronbach’s alpha coefficient of this scale in this study is 0.917, which has a good reliability level.

#### 3.3.2 Psychological security

This paper adopts the Individual Psychological Security Scale developed by Li and Yan (2007) as a measurement tool to measure the psychological security level of the respondents in the work process, with a total of five question types, such as “At work, I express my true feelings,” “At work, I do not worry that expressing my true thoughts will be detrimental to me,” “I do not worry that expressing my true thoughts will be detrimental to me,” and so on. Cronbach’s alpha coefficient of this scale in this study is 0.88, which has a good reliability level.

#### 3.3.3 Risk-taking traits

By modifying the research scales of Gomez-Mejia and Balkin (1989) and Chen and Yang (2021), this paper identifies specific measures of risk-taking traits, including four items, such as “I am willing to take risks when choosing a job or a company” and “I prefer high risk, high reward jobs to low risk, stable salary jobs.” Cronbach’s alpha coefficient of this scale in this study was 0.855, which has a good level of reliability.

#### 3.3.4 Employee bootleg innovation behavior

In this study, the scale developed by Lin and Chen (2012) containing nine items to measure bootleg innovation behavior was chosen. The scale was verified to have good reliability and validity, so this study only modified the wording of the scale appropriately according to the actual research content in order to measure employees’ bootleg innovation behavior. The scale includes such phrases as “I continue to optimize some innovative ideas without authorization from my supervisors” and “While working, I often think about how to make ideas that have been rejected better.” Cronbach’s alpha coefficient of this scale in this study was 0.926, which has a good level of reliability.

The results of the Kaiser–Meyer–Olkin (KMO) and Bartlett’s test of sphericity were analyzed. As can be seen from the result table, the KMO measure of sampling adequacy is 0.949, much higher than the standard of 0.7, indicating that there are strong correlations among the questions tested, and the level of these correlations is significantly higher than the level of partial correlation, indicating that the validity test of this study has passed the test (Table 2).

**Table 2:** Validity test.

KMO		0.949
Bartlett’s test of sphericity	Approximate chi-square	7790.953
	Degree of freedom	300
	Significance	0

## 4 Result analysis

### 4.1 Validation factor analysis

To test the discriminant validity of all variables, this study conducted a validated factor analysis using the common method biases statistical approach proposed by Hao and Lirong (2004) for the four variables involved in this study: error management climate, psychological security, risk-taking traits, and employee bootleg innovation behaviors. The results are shown in Table 3, where the four-factor model had the best goodness of fit, *χ*^2^/df = 1.331, RMSEA = 0.037, NFI = 0.906, CFI = 0.975, and TLI = 0.972, providing support for the differentiation of the four variables involved in this study.

**Table 3:** Results of validation factor analysis.

Factor	*χ*^2^/df	NFI	CFI	TLI	RMSEA
EMA, PS, RTP, BI	1.331	0.906	0.975	0.972	0.037
EMA,PS + RTP, BI	2.844	0.796	0.857	0.842	0.088
EMA + PS + RTP, BI	4.327	0.688	0.739	0.714	0.118
EMA + PS + RTP + BI	7.546	0.453	0.485	0.438	0.165

EMA, error management climate; PS, psychological security; TP, risk-taking propensity; BI, Bootleg innovation.

Common method bias refers to the artificial covariance between predictor and criterion variables due to the same data collection method (such as self-report and questionnaire) or other external factors (such as social expectations and personal tendencies) (Podsakoff et al., 2012). This bias can seriously affect the accuracy and reliability of the study results. To detect and assess the impact of common methodology bias, we used the Harman single-factor tests. As can be seen from Table 4, the first common factor explains 33.436% of the total variance, which is less than the critical value of 40% (Podsakoff et al., 2003). Therefore, there is no serious common methodology bias problem in this study.

**Table 4:** Harman’s single-factor test.

Component	Initial eigenvalues			Extraction sums of squared loadings		
	Total	% of variance	Cumulative %	Total	% of variance	Cumulative %
1	8.359	33.436	33.436	8.359	33.436	33.436
2	3.588	14.353	47.789	3.588	14.353	47.789
3	2.703	10.812	58.601	2.703	10.812	58.601
4	2.006	8.022	66.624	2.006	8.022	66.624
5	0.699	2.796	69.419			
6	0.624	2.495	71.915			
7	0.574	2.294	74.209			
8	0.555	2.220	76.430			
9	0.538	2.153	78.582			
10	0.504	2.017	80.599			
11	0.479	1.915	82.514			
12	0.461	1.842	84.356			
13	0.449	1.796	86.152			
14	0.410	1.642	87.794			
15	0.368	1.473	89.267			
16	0.347	1.390	90.657			
17	0.331	1.325	91.981			
18	0.321	1.286	93.267			
19	0.291	1.165	94.432			
20	0.275	1.098	95.530			
21	0.262	1.048	96.578			
22	0.248	0.990	97.569			
23	0.227	0.907	98.476			
24	0.199	0.798	99.273			
25	0.182	0.727	100.000			

Variance inflation factor (VIF) is a statistic that measures the severity of multicollinearity (Senaviratna and Cooray, 2019). In a multiple linear regression model, when there is a high linear correlation between two or more independent variables, it is called multicollinearity. Multicollinearity can cause the variance of regression coefficient estimators to increase, thus reducing the stability and reliability of the model. VIF value is an index used to detect this multicollinearity problem. Through collinearity diagnosis, we found that VIF values were all less than 10, indicating that there was no multicollinearity between variables (Table 5).

**Table 5:** Collinearity diagnosis.

	Tolerance	VIF
EMA	0.752	1.329
PS	0.699	1.431
RTP	0.800	1.249
Gender	0.972	1.028
Age	0.735	1.361
Education	0.872	1.147
Length of service	0.590	1.695
Enterprise size	0.570	1.755

### 4.2 Correlation analysis

As shown in Table 6, there was a significant positive correlation between the main variables in this study, with error management climate being significantly positively correlated with psychological security (*r* = 0.434, *p* < 0.01) and bootleg innovation behavior (*r* = 0.320, *p* < 0.01), and psychological security being significantly correlated with bootleg innovation behavior (*r* = 0.385, *p* < 0.01).

**Table 6:** Descriptive statistics.

		EMA	PS	BI	RTP	Gender	Age	Education	Length of service	Enterprise size
EMA	Pearson correlation	1	0.434^**^	0.320^**^	0.224^**^	−0.013	0.035	0.100	−0.031	0.055
	Sig.		0.000	0.000	0.000	0.841	0.587	0.120	0.637	0.393
PS	Pearson correlation	0.434^**^	1	0.385^**^	0.093	0.022	0.094	0.213^**^	0.017	0.111
	Sig.	0.000		0.000	0.151	0.733	0.145	0.001	0.788	0.086
BI	Pearson correlation	0.320^**^	0.385^**^	1	0.178^**^	0.000	0.000	0.082	−0.092	−0.005
	Sig.	0.000	0.000		0.005	0.998	0.998	0.207	0.154	0.935
RTP	Pearson correlation	0.224^**^	0.093	0.178^**^	1	−0.040	0.050	0.030	−0.071	0.029
	Sig.	0.000	0.151	0.005		0.536	0.443	0.639	0.274	0.653
Gender	Pearson correlation	−0.013	0.022	0.000	−0.040	1	0.039	−0.110	−0.030	0.029
	Sig.	0.841	0.733	0.998	0.536		0.542	0.087	0.646	0.658
Age	Pearson correlation	0.035	0.094	0.000	0.050	0.039	1	−0.007	0.432^**^	0.474^**^
	Sig.	0.587	0.145	0.998	0.443	0.542		0.908	0.000	0.000
Education	Pearson correlation	0.100	0.213^**^	0.082	0.030	−0.110	−0.007	1	−0.146^*^	0.107
	Sig.	0.120	0.001	0.207	0.639	0.087	0.908		0.023	0.098
Length	Pearson correlation	−0.031	0.017	−0.092	−0.071	−0.030	0.432^**^	−0.146^*^	1	0.575^**^
	Sig.	0.637	0.788	0.154	0.274	0.646	0.000	0.023		0.000
Size	Pearson correlation	0.055	0.111	−0.005	0.029	0.029	0.474^**^	0.107	0.575^**^	1
	Sig.	0.393	0.086	0.935	0.653	0.658	0.000	0.098	0.000	

**At level 0.01 (two-tailed), the correlation was significant.

*At level 0.05 (two-tailed), the correlation was significant.

### 4.3 Hypothesis testing analysis

Aggregation validity was analyzed using AVE (mean variance extraction) and CR (combined reliability). As can be seen from Table 7, the AVE of the seven common factors is greater than 0.5, the CR value is greater than 0.7, and the standardized load coefficient is greater than 0.6, indicating high polymerization validity, which indicates that the model has strong validity.

**Table 7:** Differential validity analysis.

	EMA	PS	RTP	BI
EMA	0.785			
PS	0.434	0.773		
RTP	0.224	0.093	0.773	
BI	0.32	0.385	0.178	0.767

The blue section represents CR (construct reliability), which reflects the extent to which all items within each latent variable consistently explain the latent variable. A CR value greater than 0.70 indicates that the latent variable has good construct reliability.

#### 4.3.1 Main and mediating effects

Considering the main effect and mediating effect first, H1, H2, H3, and H4 were tested using hierarchical regression: (i) Control variables (gender, age, education, length of service, and enterprise size) and independent variables (error management climate) were introduced into the regression equations, in turn, to analyze the effect of error management climate on employees’ bootleg innovation behavior. (ii) Control variables and independent variables (error management climate) are introduced sequentially to analyze the effect of error management climate on psychological security. (iii) Control variables and mediating variables (psychological security) are introduced sequentially to analyze the effect of psychological security on employees’ bootleg innovation behavior. (iv) Mediating effect, introducing control variables first, and then putting in independent variables and mediating variables to analyze the influence of error management climate and psychological security on employees’ bootleg innovation behavior. In statistics, the definition of “significant” is often associated with the level of significance and is used to determine the critical probability value for rejecting the null hypothesis in a hypothesis test. In general, the significance level is preset before the statistical test is performed, and commonly used significance level values are 0.05 or 0.01. When *p* < 0.05: The result is generally considered statistically significant; that is, there is sufficient evidence to reject the null hypothesis and believe that the difference between the sample data and the null hypothesis is not caused by random error. When *p* < 0.01: The result is considered more significant, which indicates that the difference between the sample data and the original hypothesis is more clear and the possibility of random error is lower. When *p* < 0.001, in some studies, a more stringent significance level, such as 0.001, is used, where a *p*-value less than 0.001 is considered to have a statistically significant difference. The results are shown in Table 8. The positive effect of error management climate on employees’ bootleg innovation behavior is significant (*β* = 0.444, *p* < 0.01). Thus, H1 is validated. The positive effect of error management climate on psychological security was significant (*β* = 0.297, *p* < 0.01). Thus, H2 was validated. It indicated a significant positive effect of psychological security on employees’ bootleg innovation behavior (*β* = 0.275, *p* < 0.01), and H3 was validated. Moreover, the positive effect of error management climate on employees’ bootleg innovation behavior was still significant (*β* = 0.133, *p* < 0.01) after the addition of psychological security, but the coefficient was reduced. Thus, H4 is validated, i.e., psychological security plays a partially mediating role in error management climate and employee bootleg innovation behavior. The test of mediating effect was conducted using PROCESS using the bootstrap method and by bias-correcting the non-parametric percentage bootstrap while utilizing the bias-corrected test method with repeated sampling 2000 times at a 95% confidence interval condition. According to the mediation effect judgment criteria, if the interval of the value of the variable at the 95% confidence interval does not include 0, it indicates that the mediation effect is significant. As shown in Table 9, the indirect effect of error management climate on employees’ bootleg innovation behavior through psychological security has a higher effect value in the high-level group (*β* = 0.177, 95% CI = [0.108, 0.255]) than in the low-level group (*β* = 0.072, 95% CI = [0.001, 0.146]), and the difference in the average group is significant as well (*β* = 0.072, 95% CI = [0.001, 0.146]; 95% CI = [0.071, 0.187]), indicating that psychological security mediates the relationship between error management climate and bootleg innovation behavior.

**Table 8:** Hypothesis test results.

	Psychological security			Bootleg innovative behavior
Gender	0.006	−0.02	−0.013	0.09
Age	0.019	0.004	−0.009	0.056
Academic qualifications	0.036	−0.02	−0.011	0.208**
Length of service	−0.102	−0.11	−0.086	0.015
Enterprise size	0.023	0.011	0.012	0.045
Error management climate	0.297**	0.175**	0.444**	0.133*
Risk-taking traits profile			−0.25	
Psychological security			−0.179	0.275**
Psychological security* risk-taking traits				0.141**
R^2^	0.112	0.186	0.217	0.228
△R^2^	0.089	0.162	0.183	0.204
*F*-value	4.907**	7.615**	7.133**	11.490**

*p < 0.05; **p < 0.01.

**Table 9:** Mediation effect test.

Intermediary variable	Adjustment level	Level value	Effect	BootSE	BootLLCI	BootULCI
Psychological security	Low level (−1SD)	2.792	0.072	0.035	0.01	0.146
	Average value	3.757	0.125	0.03	0.071	0.187
	High level (+1SD)	4.722	0.177	0.038	0.108	0.255

BootLLCI refers to the lower limit of the bootstrap sampling 95% interval and BootULCI refers to the upper limit of the bootstrap sampling 95% interval.

#### 4.3.2 Moderating effect of risk-taking traits

In order to explore the moderating effect of risk-taking traits on psychological security and employee piracy innovation, we designed a regression analysis study. In this study, we not only introduced control variables to ensure the accuracy of the results but also included independent variables, mediating variables, and moderating variables. Crucially, we add an interaction term between psychological security and risk-taking traits to the regression equation to examine their interaction.

The results of regression analysis showed that the interaction terms of psychological security and risk-taking traits had a significant positive effect on the employees’ bootleg innovation behavior (*β* = 0.122, *p* < 0.05). This finding clearly points out the positive moderating effect of the risk-taking trait on the relationship between psychological security and bootleg innovation behavior: when the level of risk-taking trait is higher, the positive promoting effect of psychological security on bootleg innovation behavior is more significant. In contrast, when the level of risk-taking trait is low, the positive effect is relatively weakened. This result strongly supports our hypothesis H5.

**Figure 2:**
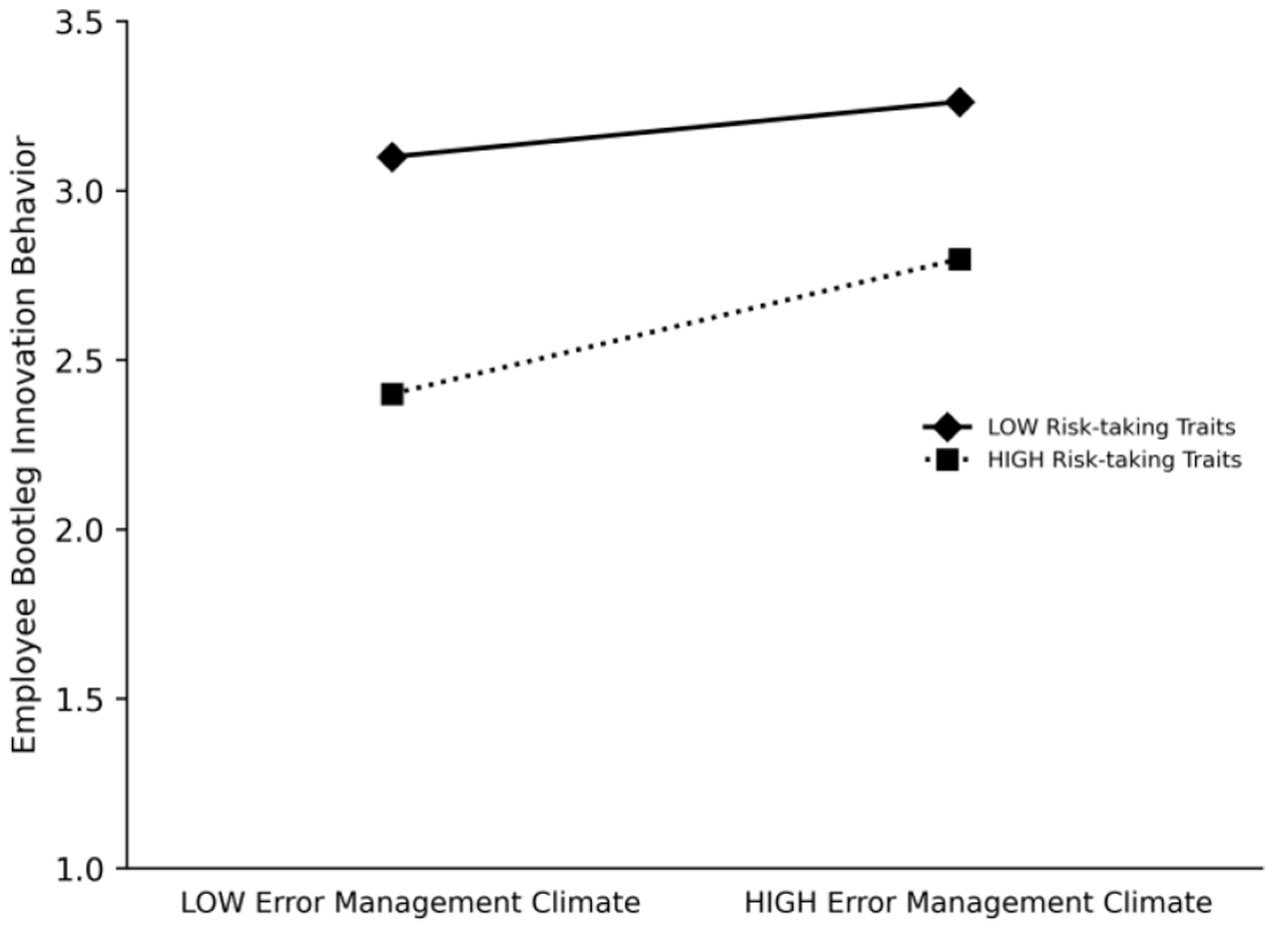
Moderating role of risk-taking traits.

In order to further visually demonstrate the moderating effect of risk-taking traits, we analyzed the moderating effect using the method of one standard deviation above the mean and one standard deviation below the mean and presented the results in Figure 2. The results showed that the moderating effect of psychological security on bootleg innovation was 0.3984 (*p* < 0.01) in the state of high-risk-taking trait and 0.1625 (*p* < 0.01) in the state of low risk-taking trait. This comparison clearly shows that psychological security has a stronger positive impact on bootleg innovation in situations with higher risk-taking traits.

## 5 Conclusion

### 5.1 Main findings and theoretical contributions

Taking the working employees of enterprise units as the research sample, this study constructed a relationship model of error management climate on employees’ bootleg innovation behavior and explored the influence mechanism of error management climate on employees’ bootleg innovation behavior, the mediating effect of psychological security, and the moderating effect of risk-taking traits. Based on the empirical test results, the following conclusions are drawn:

First, in business organizations, error management climate has a significant positive impact on employees’ bootleg innovation behavior. The more employees perceive an error management climate at work, the more their bootleg innovation behavior tends to increase accordingly. Previous research has focused on external reward and punishment systems (Abrams et al., 2018), and less on the effects of error management climate on employees’ psychological states and subsequent behavior. In a management climate that encourages acceptance of mistakes and is learning oriented, employees are more inclined to exhibit bootleg innovation behavior. This climate leads employees to believe that the organization encourages innovation and experimentation with new approaches, and therefore, they are more willing to actively explore new ideas, experiment with new methods, and take risks in order to improve work processes or product services. This study explores the results of the impact of error management climate with on-the-job employees in an enterprise unit as the research object, and the results support the theoretical view that establishing a strong error management climate can promote employees’ bootleg innovation behaviors, validate the effectiveness of the error management climate in encouraging employees to adopt bootleg innovation behavior, and expand the content of the research on error management climate.

Second, psychological security played a key mediating role between error management climate and employee bootleg innovation behavior. This finding has important implications for theoretical research. First, while past studies have tended to focus on social interaction (Edmondson and Lei, 2014), team performance, and so on (Kim et al., 2020) in exploring the influencing role of psychological security, the present study explores the formation of psychological security from the perspective of error management climate. Second, past studies have often focused on the influence of factors such as leadership style (Lu et al., 2022) and individual traits (Tenzer and Yang, 2019) on employees’ bootleg innovation behavior and seldom took employees’ intrinsic psychological states into account. In this study, we introduced psychological security as a key variable by constructing the pathway of “error management climate—psychological security—employee bootleg innovation behavior” and emphasized its role in error management climate and employee bootleg innovation behavior. By constructing the path of “error management climate—psychological security—employee bootleg innovation behavior,” this study introduces psychological security as a key variable and highlights its mediating role between error management climate and employee bootleg innovation behavior. This study not only expands the understanding of the relationship between error management climate and bootleg innovation behavior but also highlights the important role of psychological security in this process, enriching the research on the antecedent and outcome variables of psychological security.

Third, risk-taking traits positively moderated the relationship between psychological security and employees’ bootleg innovation behavior, and the interaction of higher risk-taking traits and psychological security had a significant positive effect on employees’ bootleg innovation behavior. This finding lends support to the previous hypothesis H5. Individuals high in risk-taking traits are more willing to try out new ideas and approaches, and having an environment with a high level of psychological security reduces their worries and fears, giving them more confidence and motivation to try out and implement novel and innovative ideas. This psychological security allows risk-takers to explore and take risks more freely, which promotes them to demonstrate bootleg innovation behavior more frequently, leading to more opportunities for innovation and development in the organization. The significance of this finding lies in the following: first, previous studies have mainly considered the moderating effect between psychological security and employees’ bootleg innovation behavior in terms of organizational culture variables and have rarely explored the moderating effect from an individual trait perspective; second, previous studies on risk-taking traits have mainly considered the antecedent and consequent variables of risk-taking traits and the mediating role of risk-taking traits among the relevant variables, neglecting the role of risk-taking traits on psychological security and innovation. The findings of this study not only examined the relationship between error management climate and employees’ deviant and innovative behavior as a series of influences that positively feedback to satisfy individuals’ psychological needs, and then stimulate intrinsic motivation and lead to behavior, but also deepened the research on the moderating effect of risk-taking traits on psychological security, which provides ideas for further research on psychological security in the future.

### 5.2 Practical implications

This study focuses on the relationship between error management climate and employees’ bootleg innovation behavior, which broadens the research horizon and deepens managers’ understanding of the value of error management climate. It is found that the error management climate can stimulate more employees’ bootleg innovation behavior by enhancing psychological security and promote organizational innovation and development. For enterprises, this study puts forward the following recommendations:

First, managers should encourage employees to try new ideas, accept mistakes in the innovation process, and create an atmosphere of safe innovation. Use constructive and supportive communication to deal with staff mistakes and reduce their tension; build a team culture that encourages knowledge sharing, collaboration, and mutual support and enhance employees’ confidence and motivation to innovate.

At the same time, managers should give priority to enhancing the psychological security of employees by providing support and resources, promoting teamwork, and demonstrating a leadership role with a positive innovative and experimental attitude to promote the psychological security of employees, thus encouraging piracy innovation.

In addition, managers should pay attention to the risk-taking traits of employees and provide them with appropriate challenges and opportunities to increase their self-confidence and willingness to take risks. Provide regular positive feedback and recognition for their efforts and innovative attempts to create an atmosphere that fosters risk-taking traits and promotes piracy innovation and positive risk-taking.

### 5.3 Limitations and future research

This study is devoted to exploring the mediating mechanism of psychological security between the error management climate and the employees’ bootleg innovation behavior, and in the process, we found the moderating effect of risk-taking trait on this relationship. Although the research has achieved some results through theoretical derivation and empirical analysis, it still faces some significant limitations. Specifically, this study focused on a few cities in China, which limits the general applicability of the findings and makes it difficult to determine whether the findings are equally applicable to a broader international context. In addition, the use of electronic questionnaires to collect data may lead to bias in sample selection as it may not fully cover all potential study subjects, especially those with limited digital resources or who are less willing to participate in the survey online. Therefore, in order to more comprehensively validate and extend the conclusions of this study, future studies need to span different geographic regions and combine multiple data collection methods to ensure the diversity and representativeness of the sample, thus improving the accuracy and reliability of the study. The study also overlooks critical methodological weaknesses like potential response bias in self-reported data and lack of longitudinal data to assess causality.

## Data Availability

The raw data supporting the conclusions of this article will be made available by the authors, without undue reservation.
